# Case Report: Extrapulmonary Manifestations of COVID-19 and Dengue Coinfection

**DOI:** 10.4269/ajtmh.21-0177

**Published:** 2021-06-28

**Authors:** José Manuel Reyes-Ruiz, Rosa Campuzano-Vences, Juan Fidel Osuna-Ramos, Luis Adrián De Jesús-González, María J. Pérez-Méndez, Crescencio González-González, Carlos Noe Farfan-Morales, Leticia Rivas-Tovar, Eduardo Dávila-González, Rosa María del Ángel, Aarón P. Gutiérrez-Garduño, Enrique Villegas-del Ángel, Paola Zárate-Segura, Fernando Bastida-González

**Affiliations:** 1 Unidad Médica de Alta Especialidad, Hospital de Especialidades No. 14, Centro Médico Nacional “Adolfo Ruiz Cortines”, Instituto Mexicano del Seguro Social, Veracruz, Veracruz, Mexico;; 2 Laboratorio de Biología Molecular, Laboratorio Estatal de Salud Pública del Estado de México, State of Mexico, Mexico;; 3 Department of Infectomics and Molecular Pathogenesis, Center for Research and Advanced Studies (CINVESTAV-IPN), Mexico City, Mexico;; 4 Hospital General Miguel Hidalgo y Costilla Bicentenario, State of Mexico, Mexico;; 5 Departamento de Epidemiología, Jurisdicción Sanitaria Tlanepantla ISEM, State of Mexico, Mexico;; 6 Centro Médico ABC, Mexico City, Mexico;; 7 Laboratorio de Medicina Traslacional, Escuela Superior de Medicina, Instituto Politécnico Nacional, Mexico City, Mexico

## Abstract

The risk of coronavirus disease 2019 (COVID-19) and dengue coinfection is increased in tropical countries; however, the extrapulmonary clinical manifestations have not been fully characterized. We report a 42-year-old woman whose clinical manifestations began with fever, diarrhea, headache, chest pain, myalgia, odynophagia, and arthralgia. Despite mild respiratory symptoms and normal chest computed tomography scan results, she was diagnosed with real-time reverse-transcription polymerase chain reaction (RT-PCR)-confirmed severe acute respiratory syndrome coronavirus 2 (SARS-CoV-2) infection. Because she had erythema and petechiae with a decreased platelet count, the dengue NS1 antigen and anti-dengue IgM/IgG test were performed, and the Centers for Disease Control and Prevention RT-PCR assay detected the dengue virus serotype 1 infection. Additionally, increased liver enzyme serum levels were found in the patient, who later developed hepatomegaly. Hence, the mechanism of hepatic pathology associated with SARS-CoV-2 and dengue coinfection needs further research.

## INTRODUCTION

Coronavirus disease 2019 (COVID-19) is continuing to spread worldwide.^1^ Mexico is a country with endemic arthropod-borne viral diseases such as dengue (caused by all four serotypes of dengue virus [DENV; DENV-1 to DENV-4]) and confirmed cases of COVID-19.^2^ In France and Thailand, co-circulation and evidence of coinfections among those with severe acute respiratory syndrome coronavirus 2 (SARS-CoV-2), the causative agent of COVID-19, and DENV were demonstrated.^3^^,^^4^ Because of the tropical climate and spread of SARS-CoV-2 in Mexico and other endemic areas, individuals are at risk for coinfections with dengue. Recent case reports have described similar clinical manifestations for COVID-19/dengue-coinfected patients.^3^^,^^4^ Nevertheless, there is little evidence of the extrapulmonary manifestations of SARS-CoV-2 and DENV coinfections. It is essential to recognize the clinical features of these coinfections to achieve appropriate management. We report a Mexican woman with both COVID-19 and dengue hemorrhagic fever (DHF). She experienced increased hepatic aminotransferase levels and later developed extrapulmonary clinical manifestations such as hepatomegaly.

## CASE PRESENTATION

**Figure 1.**
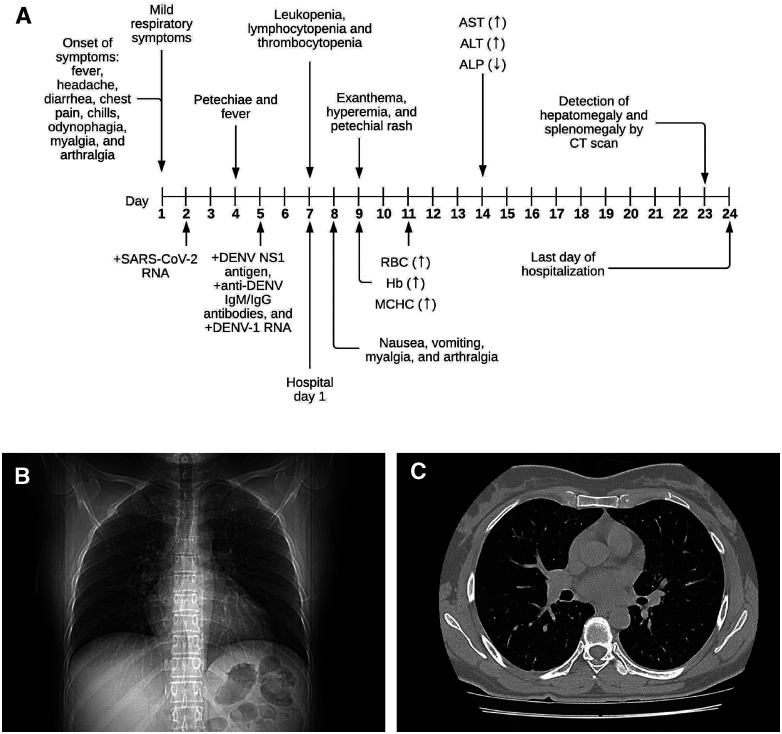
(**A**) Timeline showing the clinical manifestations and laboratory parameters for coronavirus disease 2019 (COVID-19) and dengue coinfection. (**B** and **C**) Chest computed tomography (CT) on the day of admission (day 7 after the onset of symptoms) showing no abnormality. +, positive test result; (↓) or (↑), the value was below or above the reference range, respectively; RBC, red blood cell; Hb, hemoglobin; MCHC, mean corpuscular hemoglobin concentration; AST, aspartate aminotransferase; ALT, alanine aminotransferase; ALP, alkaline phosphatase; CT, computed tomography.

**Figure 2.**
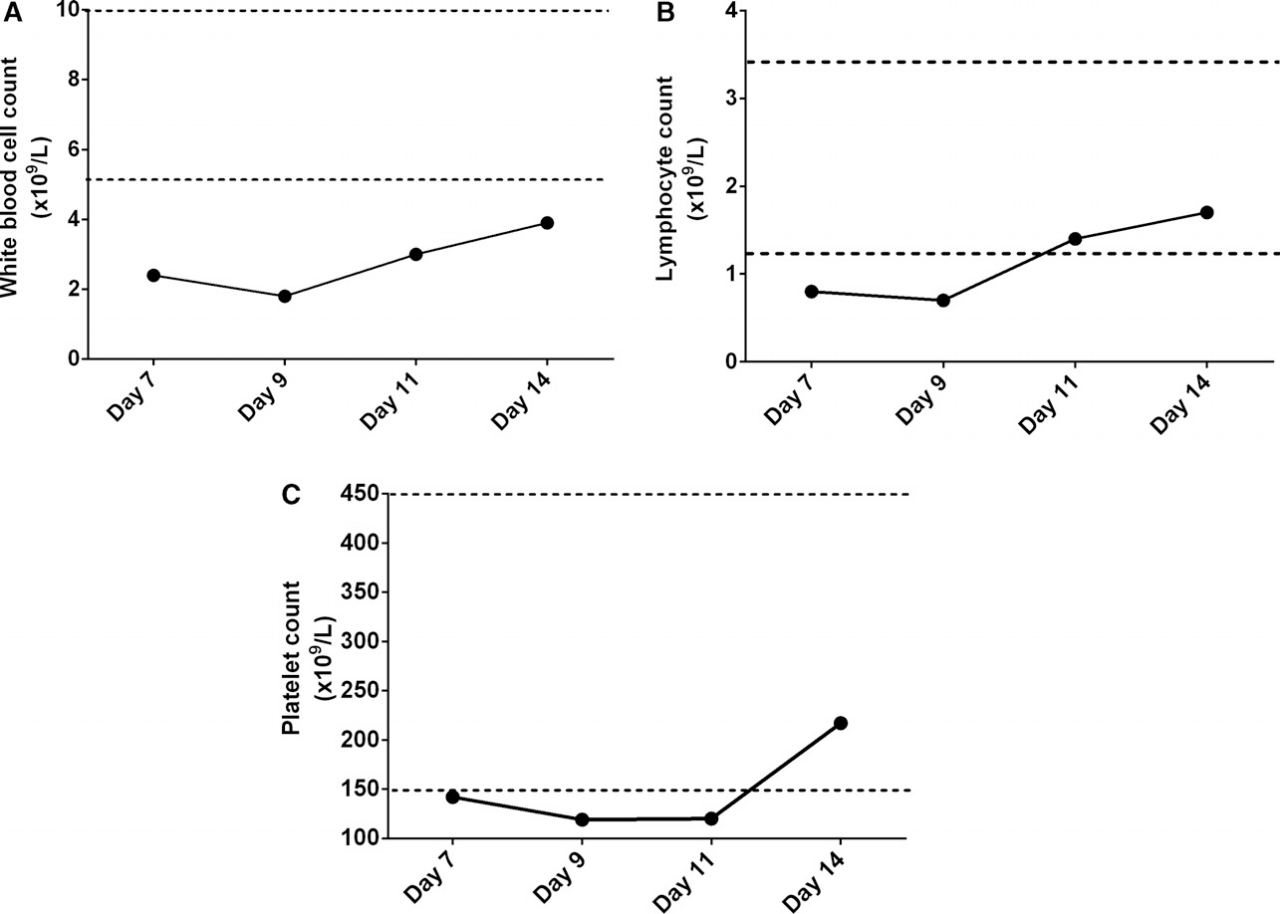
Line chart of the hematologic parameters involved in coronavirus disease 2019 (COVID-19) and dengue virus infection. The white blood cell (**A**), lymphocyte (**B**), and platelet counts (**C**) decreased on days 7 and 9 of hospitalization. However, the lymphocyte and platelet counts increased to its reference range within 11 and 14 days of hospitalization. The dotted lines represent the reference range.

On August 11 (day 1, onset of symptoms), a 42-year-old Mexican woman developed fever (nonquantified), headache, diarrhea, chest pain, chills, odynophagia, myalgia, and arthralgia. She had no comorbidity and no history of alcoholism or smoking (Figure 1A). Because she also had mild respiratory symptoms, a diagnostic test for SARS-CoV-2 was requested. On day 2 after the onset of symptoms, the nasopharyngeal and oropharyngeal swabs were analyzed using real-time reverse-transcription polymerase chain reaction (RT-PCR).^2^ The results indicated SARS-CoV-2. However, chest computed tomography (CT) showed no abnormality (Figure 1B and C). Subsequently, on day 4 after the initial symptoms, she reported general malaise, itching, low back pain, nausea, loss of appetite, and sweating, with a body temperature of 39°C. On August 14 (day 4), enlargement of the liver and spleen was not detected by palpation, but petechiae were located on her abdomen. Therefore, the patient was advised to seek an assessment for suspected DHF. It was determined that she had DENV NS1 antigens and anti-DENV IgM and IgG antibodies (SD BIOLINE dengue Duo, Standard Diagnostic, Inc., Kyonggi-do, Korea). The DENV-1 infection was confirmed by the Centers for Disease Control and Prevention DENV-1-4 RT-PCR assay (5 days after the onset of symptoms).^5^ Consequently, on day 7 after the initial symptoms (hospital day 1), she was admitted to the general hospital in Mexico for surveillance of COVID-19 and dengue coinfection. She was treated with azithromycin, ibuprofen for 4 days, and a single ivermectin dose. On admission (August 17), her vital signs were as follows: temperature, 37°C; 97% oxygen saturation; pulse, 88 beats per minute; respiratory rate, 20 breaths per minute; and blood pressure, 130/90 mmHg. Moreover, laboratory studies performed as part of the follow-up (hospital day 1) showed that her leukocyte, lymphocyte, and platelet counts were below the normal ranges (Figure 2A–C).

During the subsequent hospitalization day (days 7–24 after the onset of symptoms), her vital signs remained stable and she was afebrile. Nonetheless, during the second day of hospitalization (day 8 after initial symptoms), she experienced vomiting, myalgia, arthralgia, and nausea. Physical examination (hospital day 3; day 9 after the onset of symptoms) indicated hyperemia on the patient’s face. Additionally, exanthema and generalized confluent petechial rash confirmed DHF. Ciprofloxacin (500 mg) and loratadine tablets (10 mg) were administered orally every 12 hours. Ibuprofen was discontinued and paracetamol (500 mg every 8 hours) was administered. The patient’s condition and symptoms improved significantly after medical treatment. On hospital day 5, her vital signs were normal, with oxygen saturation maintained above 97%. However, she experienced itching on the palms and soles. She was administered chlorpheniramine (4 mg administered orally every 8 hours) for 3 days. Laboratory findings showed that the red blood cell level, hemoglobin level, mean corpuscular hemoglobin concentration, and mean platelet volume were above the normal ranges (hospital days 3 and 5; days 9 and 11 after the initial symptoms) (Table 1). On day 14 after the onset of symptoms, she was afebrile and had discreet erythema. Blood test results revealed high levels of aspartate aminotransferase (AST) and alanine aminotransferase (ALT) (Table 1),^6^^,^^7^ suggesting hepatic injury.

**Table 1:** Laboratory results during SARS-CoV-2 and DENV-1 coinfection in the patient

Variable	Reference range	7th day after the onset of symptoms (hospital day 1)	9th day after the onset of symptoms (hospital day 3)	11th day after the onset of symptoms (hospital day 5)	14th day after the onset of symptoms (hospital day 8)
Lymphocyte ratio (%)	21–48	32.2	36.5	48.1	42.4
Monocyte count (x109/L)	0–0.7	0.2	0.1	0.2	0.3
Monocyte ratio (%)	2–8	8.2 (↑)	7	7.6	7.6
Granulocytes count (x109/L)	2–7	1.4 (↓)	1 (↓)	1.4 (↓)	1.9 (↓)
Granulocytes ratio (%)	50–70	59.6	56.5	44.3 (↓)	50
Red blood cells (x106/µL)	3.5–5	4.83	5.17 (↑)	5.09 (↑)	4.97
Hemoglobin (g/dL)	11.5–16.5	16	16.9 (↑)	16.8 (↑)	16.3
Hematocrit (%)	37–54	42.6	45.2	44.7	43.4
MCV (fL)	80–100	88.3	87.5	87.7	87.3
MCH (pg)	27–34	33	32.7	32.9	32.9
MCHC (g/dL)	33–35	37.4 (↑)	37.4 (↑)	37.6 (↑)	37.6 (↑)
RDW-CV (%)	11.5–14.5	12.4	12.3	12.2	12
RDW-SD (fL)	35–56	40.2	39.3	39.3	38.4
Mean platelet volume (fL)	7.4–11	12.2 (↑)	11.6 (↑)	12.3 (↑)	10.6
Plateletcrit (%)	0.19–0.36	0.173 (↓)	0.138 (↓)	0.147 (↓)	0.230
Platelet distribution width (%)	15.5–17.1	16	16.5	16.2	16.5
Albumin (g/dL)	3.4–5	–	–	–	4.1
Globulin (g/dL)	2–4	–	–	–	3.8
Albumin/globulin ratio	1.1–1.9	–	–	–	1.08 (↓)
Total bilirubin (mg/dL)	0–1	–	–	–	0.5
Direct bilirubin (mg/dL)	0–0.3	–	–	–	0.1
Indirect bilirubin (mg/dL)	0–0.7	–	–	–	0.4
Aspartate aminotransferase (U/L)	5–34	–	–	–	48 (↑)
Alanine aminotransferase (U/L)	5–34	–	–	–	75 (↑)
Alkaline phosphatase (U/L)	50–136	–	–	–	32 (↓)

MCV = mean corpuscular volume; MCH = mean corpuscular hemoglobin; MCHC = mean corpuscular hemoglobin concentration; RDW-CV = red cell volume distribution width-coefficient of variation; RDW-SD = red cell volume distribution width-standard deviation. The value in the patient was below (↓) or above (↑) the reference range.

**Figure 3.**
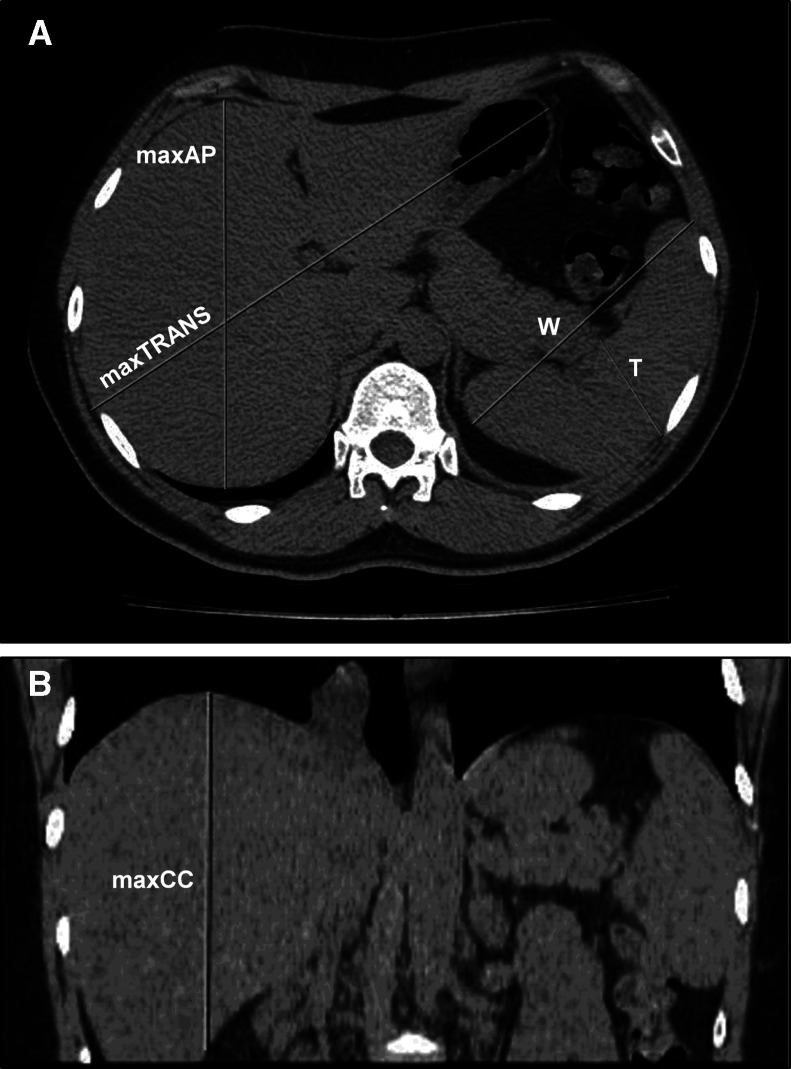
Detection of hepatomegaly and splenomegaly using computed tomography (CT) scan. CT abdomen axial (**A**) and coronal (**B**) sections showing the maximum anteroposterior (maxAP = 15.84 cm), transverse (maxTRANS = 22.27 cm), and craniocaudal distances (maxCC = 13.41 cm) of the liver and the maximum splenic width (W = 12.15 cm) and thickness (T = 4.56 cm). The splenic craniocaudal distance (CC = 10 cm) was evaluated using the cranial-most and caudal-most ends of the spleen on the axial sections. Volumes of 1,819.4 cm^3^ and 554.04 cm^3^ for the liver and spleen, respectively, were calculated. This figure appears in color at www.ajtmh.org.

On day 23 after the initial symptoms (day 17 of hospitalization), follow-up chest CT imaging showed no pulmonary infiltration. Nevertheless, marked enlargement of the liver and spleen was observed (Figure 3A and B). The patient was discharged from the hospital the next day (hospital day 18; day 24 after the onset of symptoms) and instructed to undergo liver function tests within 1 week. She was considered ineligible to receive alcohol or any acetaminophen products. Unfortunately, she was lost to follow-up.

## DISCUSSION

We report an autochthonous case of SARS-CoV-2 and DENV coinfection in a woman during the current COVID-19 epidemic in Mexico. The patient initially presented nonspecific signs and symptoms. She also had mild respiratory symptoms and chest pain without pneumonia on CT imaging. The positive RT-PCR test results for SARS-CoV-2 confirmed mild COVID-19.^8^ On day 4 after the onset of symptoms, she presented with petechiae located on the abdomen. This sign alerted the clinicians and raised clinical suspicions for DHF because she lives in a dengue-endemic area. The World Health Organization defined DHF as fever, thrombocytopenia, and bleeding manifestations.^7^^,^^9^ These characteristics were observed in our patient. The presence of DENV IgM antibodies (day 5 after the initial symptoms), which was detected on days 3 to 5 of illness,^9^ suggested that the infection was recent. Patients infected with SARS-CoV-2 or DENV develop symptoms within 4 to 10 days (incubation period)^1^^,^^9^; therefore, we suggest that both infections could have started at almost the same time, thus generating the viral coinfection observed with this clinical case.

Although the skin manifestations are a standard feature of DHF, they can be found with COVID-19.^10^ Because both DENV and SARS-CoV-2 infections are similar, none of the clinical features was atypical for DHF. Furthermore, DENV with superimposed asymptomatic COVID-19 infection could be considered. Therefore, recognizing or ruling out this coinfection may be difficult for clinicians, and molecular and serological tests are necessary for diagnostic accuracy. COVID-19 and dengue share similar laboratory features, such as lymphopenia, leukopenia, thrombocytopenia, and elevated transaminase level,^1^^,^^11^^,^^12^ as observed in this case. Coagulopathy, which is induced by the inflammatory response, is an indicator of DHF and COVID-19.^10^^,^^13^ However, coagulation parameters and inflammatory biomarkers such as D-dimer, prothrombin time, partial thromboplastin time, fibrinogen, C-reactive protein, and ferritin^10^^,^^13^ were not analyzed.

Patients with COVID-19 have evidence of hepatocellular injury and liver function abnormalities.^6^^,^^13^ Bloom et al. reported high AST and ALT levels for 93% (54/60 patients) of patients with COVID-19.^6^ Furthermore, other studies have reported increased ALT and AST levels for 14% to 53% of COVID-19 patients.^14^ In most studies, increased AST and ALT levels have been detected in 63% to 97% and 45% to 96% of DENV-infected patients, respectively.^7^ This clinical feature is related to hepatic injury.^7^ For our case, liver function tests revealed increased AST (48 U/liter) and ALT (75 U/liter) levels. These findings are similar to the test results of a 57-year-old woman with COVID-19 but negative for dengue according to RT-PCR^12^ from Singapore who had normal chest radiography results and thrombocytopenia levels, as in our case. Alkaline phosphatase and total bilirubin levels were not increased in our case; however, they were increased in other cases of COVID-19.^6^

Our patient was diagnosed with hepatosplenomegaly using CT imaging. A recent study indicated that the level of angiotensin-converting enzyme receptor II, which is the main host cell receptor of SARS-CoV-2, was highly expressed in bile duct cells.^15^ Moreover, SARS-CoV-2 RNA has been detected in the liver and spleen.^16^

Increased serum aminotransferase levels are associated with COVID-19 and dengue disease severity and hepatomegaly.^6^^,^^7^ Halsey et al. reported the frequency of hepatomegaly (1.1%; 20/1,716 patients) and splenomegaly (0.4%; 7/1,716 patients) in DENV-infected patients.^11^ These findings for patients from four Latin American countries are different from the results analyzed by Samanta and Sharma.^7^ The frequency of hepatomegaly was 4% to 52% for DENV-infected patients in hospitals in two Asian countries according to their report.^7^ The frequency of organomegaly for patients with COVID-19 has not been fully investigated. However, liver injury, hepatomegaly, and splenomegaly have been reported during infection.^16^^–^^18^ There is a possible association between elevated transaminases, organomegaly, SARS-CoV-2 infection, and DENV infection.

Regarding SARS-CoV-2 and DENV coinfection, the most frequent extrapulmonary manifestations include lymphopenia, leukopenia, and thrombocytopenia.^3^^,^^4^^,^^17^ All these manifestations, including increased AST and ALT levels,^3^^,^^4^^,^^17^ were observed in our case. Splenomegaly was detected with a case of DENV and COVID-19 coinfection.^17^ Nevertheless, hepatomegaly has not been observed in patients with SARS-CoV-2/DENV-1 or DENV-2 coinfection.^3^^,^^4^ We hypothesized that the cell tropism of both viruses could lead to liver injury.^7^^,^^15^

Hepatic steatosis, nonalcoholic steatohepatitis, malnutrition, hepatitis viral, alcoholism, metabolic disorders, and cirrhosis as other hepatomegaly causes were ruled out. These results were obtained by analyzing the medical history and social history of the patient and laboratory test results. To investigate the possible effects on the liver induced by the medications administered to the patient, research using the LiverTox website (livertox.nih.gov; https://www.ncbi.nlm.nih.gov/books/NBK547852/) was performed. Because the effects of the previously used medicines on the liver were initially ruled out, SARS-CoV-2-induced liver damage through direct and indirect interactions inducing elevated transaminases^14^^,^^18^ was considered.

In conclusion, this report confirmed that coinfection with DENV and SARS-CoV-2 could occur on both sides of the Pacific Ocean. However, whether organomegaly is a direct result of dengue and COVID-19 coinfection requires further study.
